# Minimal residual disease status is the prognostic determinant following high‐dose treatment for patients with multiple myeloma

**DOI:** 10.1002/cam4.6640

**Published:** 2023-11-03

**Authors:** Hareth Nahi, Gabriel Afram, Katarina Uttervall, Sandra Lockmer, Love Tätting, Gösta Gahrton, Muhammad Kashif, Evren Alici, Olga Stromberg, Monika Klimkowska, Johan Lund

**Affiliations:** ^1^ Department of Medicine Institution for biomedicine and clinical science Linköping Sweden; ^2^ Center for Hematology and Regenerative Medicine, Department of Medicine, Huddinge Karolinska Institutet Stockholm Sweden; ^3^ Department of Medicine Stockholm Sweden

**Keywords:** minimum residual disease, multiple myeloma prognosis

## Abstract

**Background:**

The presence of minimal residual disease (MRD+) following autologous stem cell transplantation (ASCT) in multiple myeloma represents a poor prognostic factor for progression‐free survival (PFS) and overall survival (OS).

**Methods:**

At our department, we recommend lenalidomide maintenance for patients who are MRD+ after ASCT, while MRD‐negative (MRD−) patients, after information about the national guidelines, were not advised to follow this regimen.

**Results:**

Out of the total 228 patients, 175 received ASCT following first‐line induction (MRD− 92 (53%), MRD+ 83 (47%), at 2 months post‐ASCT), while 53 underwent ASCT after second‐line treatment (MRD− 27 (51%), MRD+ 26 (49%), at the same time point). Comparatively, MRD− patients who did not receive maintenance demonstrated better OS than MRD+ patients who received upfront ASCT and maintenance treatment (96% vs. 86%, *p* = 0.030, at 3 years). However, nonsignificant difference was found in PFS (76% vs. 62%, at 3 years). Furthermore, second‐line ASCT, MRD− non‐maintained patients exhibited significantly better PFS than MRD+ (71% vs. 27%, *p* > 0.001, at 3 years). However, OS was better but nonsignificant (96% vs. 76%, at 3 years). Fluorescence in situ hybridization (FISH) analysis was performed on 141 out of the 228 patients. Of these, 85 (60%) patients were deemed standard risk (SR), and 56 (40%) were classified as high risk (HR). In the SR cohort, MRD− patients exhibited better PFS and OS than MRD+ patients (71% vs. 59% and 100% vs. 85%, respectively). In the HR cohort, the MRD− patients showed superior PFS but similar OS compared to MRD+ patients (66% vs. 42% and 81% vs. 80%, respectively).

**Conclusions:**

Our results indicate that being MRD− is a more crucial prognostic factor for the 3‐year PFS and OS than the presence of high‐risk cytogenetic markers or undergoing maintenance treatment. The latter appears insufficient, particularly for MRD+ patients following ASCT in the second‐line setting, suggesting that these patients may require a more intensive treatment approach.

## 1 INTRODUCTION

High‐dose therapy with melphalan, followed by autologous stem cell transplantation (ASCT), is the standard of care (SOC) for transplant‐eligible patients with multiple myeloma (MM). This approach significantly extends median OS from less than 3 years to more than 5 years for MM patients, as compared to those not treated with ASCT.^1^, ^2^ MM remains a therapeutic challenge, necessitating the constant evolution of treatment strategies. Despite the introduction of several novel therapeutic agents for MM, as an induction, over the past two decades, ASCT consolidation treatment still significantly benefits both PFS and OS in eligible patients.^3^, ^4^

With advances in treatment, it is imperative to continuously assess the role of existing strategies in light of new options. Over the past decade, randomized trials have shown that maintenance treatment after ASCT prolongs PFS, regardless of prior anti‐MM therapy.^5^, ^6^, ^7^, ^8^ Lenalidomide, a frequently used maintenance drug, has shown beneficial effects on PFS and OS. Several studies and a meta‐analysis corroborate this effect.^3^, ^4^, ^9^, ^10^ Although maintenance treatment is now considered SOC and is recommended in multiple guidelines, concerns persist regarding the long‐term consequences, such as secondary primary malignancies, fatigue, and other side effects.

As the treatment landscape for MM expands, so does the need for improved methods of monitoring disease progression and response to treatment. This has led to an increased proportion of patients achieving complete remission (CR) and stringent CR. A newly defined response category, minimal residual disease (MRD), has become vital for predicting PFS and OS. MRD, determined by next‐generation flow (NGF) or next‐generation sequencing (NGS), is classified as either MRD+ or MRD−. An MRD+ response to initial treatment is an adverse prognostic factor for PFS and OS, while an MRD− response is associated with better outcomes. The optimal outcome is linked with sustained MRD negativity.^11^

In our single‐center study, we aim to provide a comprehensive analysis of the impact of MRD status on the effectiveness of maintenance treatment with lenalidomide. Maintenance treatment with lenalidomide was administered to MRD+ patients following ASCT in the first or second line, while MRD− patients did not receive maintenance. This study identified and analyzed a cohort of patients who underwent ASCT treatment in the first or second line and had a documented MRD status. Understanding the interplay between MRD status and maintenance treatment could help optimize therapeutic strategies for MM.

## 2 MATERIALS AND METHODS

### 2.1 Study population and design

This research is a retrospective cohort study comprising all patients who received high‐dose melphalan (HDM) and high‐dose therapy (HDT) in the first or second line with documented minimal residual disease (MRD). We included all 228 patients diagnosed with MM (excluding plasma cell leukemia, solitary plasmacytoma, and amyloidosis) eligible for ASCT in the first or second line, treated at Karolinska University Hospital from January 2018 to November 2022. Clinical data were obtained from the hospital's electronic medical records. If patients had died after data collection, the date of death was acquired from the Swedish national death registry. The study was approved by the Ethics Committee in Stockholm (EPN 2014/526‐31/3 and 2015/973‐32). Data such as age, sex, type of myeloma, and laboratory measurements (including serum M‐protein, urine M‐protein, calcium, hemoglobin (Hb), β2‐microglobulin (b2m), albumin, creatinine, and presence of bone lesions assessed by low‐dose computed tomography) were collected for each patient.

Post‐ASCT, patients were prescribed maintenance treatment based on their MRD status, as per institutional guidelines and after receiving information on the actual national guidelines for MM treatment. MRD+ patients received lenalidomide 25 mg every other day until disease progression or the emergence of unacceptable side effects or adverse events. The median time on maintenance was 18 months (range 2–36 months). Discontinuation beyond 18 months was primarily due to the physician's discretion. Conversely, MRD− patients were not given any maintenance treatment. MRD status was assessed 2 months post‐ASCT. Patient follow‐up was conducted for PFS and OS.

### 2.2 Fluorescent in situ hybridization

Fluorescent in situ hybridization was performed as earlier described,^12^ using probe sets targeting 1q21, 17p13.1, and translocations t(4;14) (p16.3;q32.3), t(14;16)(q32.3;q23). Hybridization and signal detection followed the manufacturer's protocols. Spot counting and analysis were performed using the Olympus microscope BX60. For each probe set, 200 nuclei were evaluated. A threshold of 10% was applied as the cutoff for numerical aberrations and deletions, except for del(17p), where a 60% threshold was used. Patients were classified as high risk (HR) by FISH if they exhibited any of the aforementioned aberrations. Patients with none of these aberrations were classified as standard risk (SR). A sub‐analysis was conducted on patients harboring two or more of the HR aberrations, referred to as double hit (DT).

### 2.3 Treatments

The majority of the patients underwent induction treatment with VRD. Bortezomib (V) was administered in 3‐week cycles at a dose of 1.3 mg/m^2^ subcutaneously on days 1, 4, 8, and 11. Lenalidomide was dosed at 25 mg every other day continuously or on days 1–14 (in five patients), with the dosage adjusted according to kidney function, if needed. Dexamethasone was administered orally at 20 mg per day on days 1, 2, 4, 5, 8, 9, 11, and 12. A median of 4 induction cycles (range 3–8) was given before ASCT.

During 2018 and early 2019, VCD (Bortezomib as above, cyclophosphamide at 1000 mg/m^2^ on day one of each cycle, and Dexamethasone as above) was used as induction treatment, resulting in seven patients (3%) of the current cohort receiving VCD. The induction treatment aimed for complete remission (CR), but at least a very good partial response (VGPR) was deemed acceptable before ASCT. All patients were administered Melphalan 200 mg/m^2^ IV within 2–4 weeks of stem cell harvest (after the administration of cyclophosphamide and G‐CSF), which was followed by ASCT. No consolidation treatment was administered.

### 2.4 Minimal residual disease

The MRD status in bone marrow samples was evaluated using flow cytometry, utilizing the 2‐tube, eight‐color MM‐MRD panel by EuroFlow and a 10‐color BD FACSLyric™ cytometer (BD Biosciences), as per standard guidelines (Flores‐Montero 2017, Arroz 2016). MRD negativity was defined as the absence of myeloma cells in 100,000 analyzed bone marrow cells (10^−5^).^13^, ^14^

### 2.5 Statistical calculations and data management

Progression‐free survival and OS were estimated using the Kaplan–Meier method and compared using the log‐rank test. All figures were reported with log‐rank *p* values. Predictors for PFS and OS were evaluated using Cox regression models to estimate hazard ratios (HR). First, univariate risk factors were analyzed, and those significant were included in the subsequent multivariate model. All *p* values were two‐tailed. Analyses were conducted using SPSS software (IBM Corp) and programming language R.

## 3 RESULTS

### 3.1 Patients

Out of 242 patients, five were excluded due to nonresponse and eventual participation in clinical trials, four because of nonresponse and failure to proceed to ASCT, three MRD− patients received maintenance, and two were maintained with antiCD38. Of the remaining 228 patients, 118 (54%) were MRD+ and received maintenance treatment, while 110 (46%) tested negative for MRD, and hence, no maintenance was provided. FISH data were available for 141 patients; 85 (60%) were SR, and 56 (40%) were HR. The median follow‐up time for the entire cohort was 24 months. The median age at ASCT was 63 years and 60 years for the MRD+ and MRD− patients, respectively, *p* = 0.026. No statistical difference was noted in patient characteristics, other than age (Table 1A).

**TABLE 1A:** Patient characteristics, all patients, comparing factors at diagnosis between the minimal residual disease (MRD)+ and MRD− populations.

	All patients	MRD+	MRD−	*p* Value
*n* (%)	228 (100)	118 (52)	110 (48)	–
Gender: male no (%)	128 (56)	72 (61)	56 (51)	0.160
Age, median (range)	62 (35–76)	63 (35–76)	60 (36–75)	0.026
Heavy chain type				
IgG, no (%)	141 (62)	77 (65)	64 (58)	0.271
IgA, no (%)	34 (15)	16 (14)	18 (16)	0.683
BJ, no (%)	25 (11)	16 (14)	9 (8)	0.277
Other, no (%)	6 (3)	2 (1)	4 (4)	0.432
Unknown, no (%)	22 (9)	7 (6)	15 (14)	0.081
Light chain type				
Kappa, no (%)	140 (61)	71 (60)	69 (63)	0.795
Lambda, no (%)	65 (29)	39 (33)	26 (24)	0.154
Unknown, no (%)	23 (10)	8 (7)	15 (13)	0.134
Bone lesion, (%)	86	81	89	0.183
B2Micro, mean (range)	4.9 (1.5–38)	4.5 (1.5–31)	4.5 (1.6–38)	0.537
Hb, mean (range)	113 (64–170)	113 (64–168)	113 (74–170)	0.845
Albumin, mean (range)	33 (15–45)	33 (15–45)	32 (3–45)	0.542
Creatine, mean (range)	120 (38–1175)	107 (38–595)	134 (38–1175)	0.187
Calcium, mean (range)	2.4 (2.1–4.9)	2.4 (2.1–3.7)	2.5 (2.1–4.9)	0.230
Risk by FISH, *n* (%)	141 (100)	78 (100)	63 (100)	0.425
SR	85 (60)	50 (64)	35 (56)	
HR	56 (40)	28 (36)	28 (44)	
DH	20 (14)^a^	10 (13)^a^	10 (16)^a^	

Abbreviations: DH, double hit; FISH, fluorescence in situ hybridization; HR, high risk; SR, standard risk.

^a^ The percent of the DH is given as of the total patients analyzed with FISH, these patients are also included in the HR group.

Among 175 patients who received ASCT in the first line, 92 (53%) were MRD+ and 83 (47%) were MRD−. For the second line, out of 53 patients treated with ASCT, 26 (49%) and 27 (51%) were MRD+ and MRD−, respectively. Patient characteristics for first‐ and second‐line treatments with ASCT are summarized in Table 1B. As expected, first‐line ASCT patients were younger than second‐line patients, with median ages of 61 (35–75) and 64 (39–76) years, respectively (*p* = 0.146). In the first‐line ASCT group, the median s‐calcium was 2.4 and 2.5 mmol/L for the MRD+ and MRD− population, respectively (*p* = 0.022). No other major differences were noted.

**TABLE 1B:** Patients characteristics at first‐ and second‐line high‐dose therapy, comparing factors at diagnosis between the MRD+ and MRD− populations.

	All patients	First‐line HDT				Second‐line‐HDT			
		All	MRD+	MRD−	*p* Value	All	MRD+	MRD−	*p* Value
*n* (%)	228	175 (100)	92 (53)	83 (47)	–	53 (100)	26 (49)	27 (51)	–
Gender: male no(%)	128 (56)	101 (58)	55 (54)	46 (42)	0.667	27 (51)	17 (63)	10 (37)	0.074
Age, median (range)	62 (35–76)	61 (35–75)	62 (35–72)	60 (36–75)	0.071	64 (39–76)	64 (44–76)	62 (36–72)	0.146
Heavy chain type									
IgG, no (%)	141 (62)	108 (62)	62 (67)	46 (55)	0.141	33 (62)	15 (58)	18 (67)	0.911
IgA, no (%)	34 (15)	26 (15)	10 (11)	16 (19)	0.177	8 (15)	6 (23)	2 (7)	0.141
BJ, no (%)	25 (11)	18 (10)	12 (13)	6 (7)	0.310	7 (13)	4 (15)	3 (11)	0.704
Other, no (%)	6 (3)	6 (3)	2 (2)	4 (5)	0.425	0	0	0	‐
Unknown, no (%)	22 (9)	17 (10)	6 (8)	11 (13)	0.213	5 (10)	1 (4)	4 (15)	0.351
Light chain type									
Kappa, no (%)	140 (61)	102 (53)	51 (22)	51 (22)	0.515	38 (17)	20 (9)	18 (8)	0.600
Lambda, no (%)	65 (29)	54 (24)	33 (14.5)	21 (9.2)	0.178	11 (5)	6 (3)	5 (2)	0.944
Unknown, no (%)	23 (10)	19 (8)	8 (3.5)	11 (5)	0.469	4 (2)	0	4 (2)	0.111
Bone lesion, (%)	86	81	81	81	0.101	89	83	94	1.000
B2Micro, mean (range)	4.9 (1.5–38)	4.7 (1.5–38)	4.5 (1.5–18)	4.8 (1.6–38)	0.806	5.6 (1.7–31)	7 (2–31)	3.4 (1.7–6.2)	0.153
Hb, mean (range)	113 (64–170)	114 (64–170)	113 (64–168)	114 (77–170)	0.921	111 (70–143)	110 (70–137)	112 (74–143)	0.792
Albumin, mean (range)	33 (15–45)	33 (15–45)	33 (17–45)	32 (15–45)	0.369	32 (15–44)	31 (15–44)	32 (17–41)	0.671
Creatine, mean (range)	120 (38–1175)	124 (38–1175)	105 (47–595)	144 (38–1175)	0.116	104 (38–335)	116 (38–335)	92 (47–212)	0.337
Calcium, mean (range)	2.4 (2.1–4.9)	2.4 (2.1–4.9)	2.4 (2.1–2.9)	2.5 (2.1–4.9)	0.022	2.5 (2.1–3.7)	2.7 (2.2–3.7)	2.4 (2.1–2.8)	0.097
Risk by FISH, *n* (%)	141 (100)	110 (100)	61 (100)	49 (100)	0.613	31 (100)	18 (100)	13 (100)	0.611
SR	85 (60)	60 (55)	36 (59)	24 (49)		25 (77)	15 (83)	10 (77)	
HR	56 (40)	50 (45)	25 (41)	25 (51)		6 (23)	3 (17)	3 (23)	
DH	20 (14)^a^	19 (17)^a^	9 (15)^a^	10 (22)^a^		1 (3)^a^	1 (6)^a^	0	

Abbreviations: DH, double hit; FISH, fluorescence in situ hybridization; HR, high risk; SR, standard risk.

^a^ The percent of the DH is given as of the total patients analyzed with FISH, these patients are also included in the HR group.

### 3.2 Progression‐free survival

**FIGURE 1:**
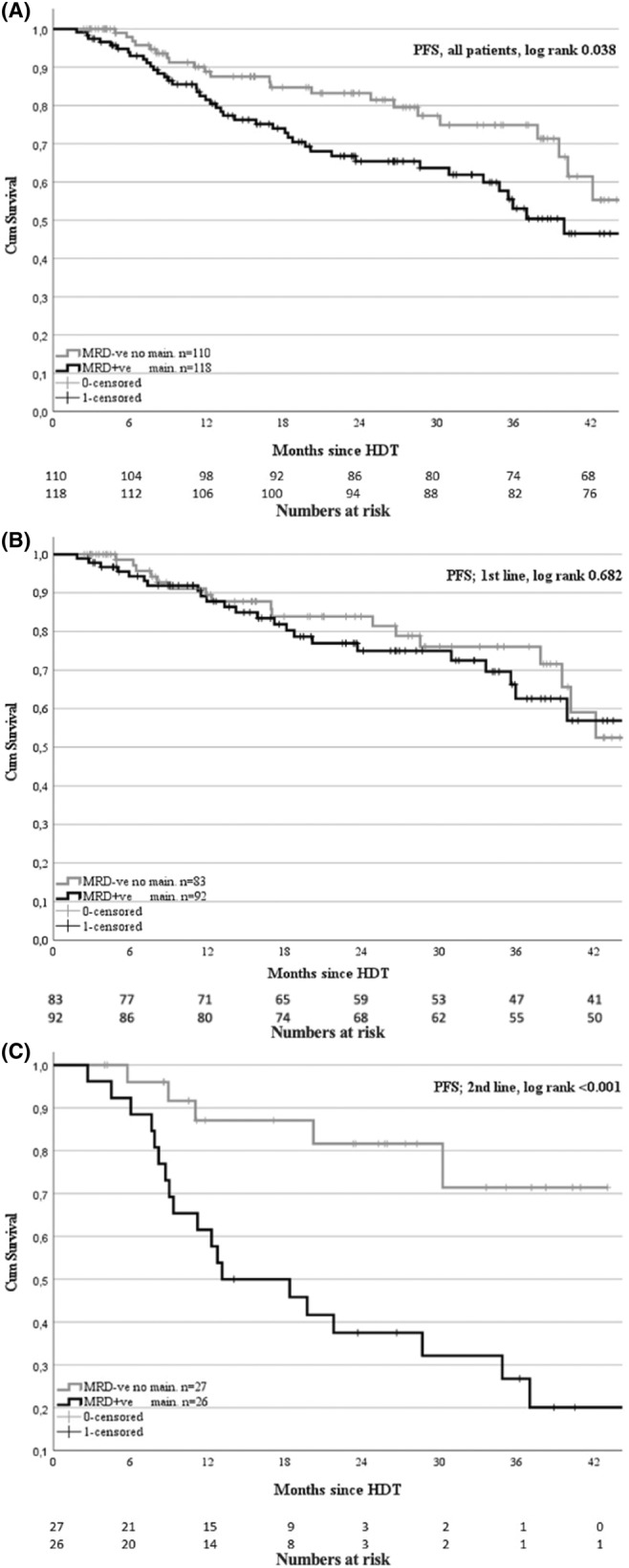
Analysis of progression‐free survival (PFS) in multiple myeloma patients grouped by minimal residual disease (MRD) status. Panel 1A; Kaplan–Meier curves of PFS for all patient grouped by MRD status. Panel 1B; PFS for the first‐line autologous stem cell transplantation (ASCT) patient grouped by MRD status. Panel 1C; PFS for the second‐line ASCT patient grouped by MRD status.

When comparing overall PFS at 3 years, MRD+ patients (52%) had significantly poorer outcomes despite receiving maintenance treatment, as compared to MRD− patients (75%), who did not receive maintenance (*p* value = 0.038, Figure 1A). Looking at SR and HR patients, the 3‐year PFS was 59% versus 71% and 42% versus 66% for MRD+ and MRD−, respectively, but these differences did not reach statistical significance. Similarly, for patients classified as double hit (DH), the 3‐year PFS was 42% and 78% for MRD+ and MRD−, respectively; however, this difference was also not statistically significant (Table 2).

**TABLE 2:** The 3‐years progression‐free survival (PFS) and overall survival (OS) for all, first‐ and second‐line high‐dose therapy (HDT).

	MRD+ (%)	MRD− (%)	*p* Value	MRD + HR (%)	MRD − HR (%)	*p* Value	MRD + SR (%)	MRD − SR (%)	*p* Value
PFS (%) at 3‐years									
All	50	75	0.038	42	66	0.450	59	71	0.235
First line	62	76	0.682	41	81	0.401	70	70	0.742
Second line	27	71	<0.001	–^a^	–^a^	–	28	83	0.051
OS (%) at 3‐years									
All	86	95	0.030	80	81	0.804	85	100	0.038
First line	90	96	0.244	78	85	0.445	88	100	0.152
Second line	75	95	0.057	–^a^	–^a^	–	80	100	0.211

^a^ Only six patients were classified as HR in the second‐line HDT, three were minimal residual disease (MRD)+, and three were MRD−.

For patients treated with ASCT in the first line, no significant difference was found between the 3‐year PFS of MRD+ and MRD− patients, 62% and 76%, respectively (Figure 1B). Interestingly, in the HR risk group, the 3‐year PFS was significantly poorer in MRD+ patients (41%) than in MRD− patients (81%).

In the second‐line ASCT group, the 3‐year PFS was significantly different, being 27% for MRD+ and 71% for MRD− patients (*p* < 0.001, Figure 1C). The 3‐year PFS for the SR patients showed a similar trend, being 28% and 83% for MRD+ and MRD−, respectively (*p* = 0.051). However, no calculations for HR and DH were performed due to insufficient patient numbers (Table 2).

We performed the Cox regression for the confounding factors age, gender, heavy/light chain, bone lesion, calcium, albumin, beta2microgloubilin, hemoglobin, creatinine, and high risk by FISH for PFS. None of these factors affect the difference PFS in any subgroup.

### 3.3 Overall survival

**FIGURE 2:**
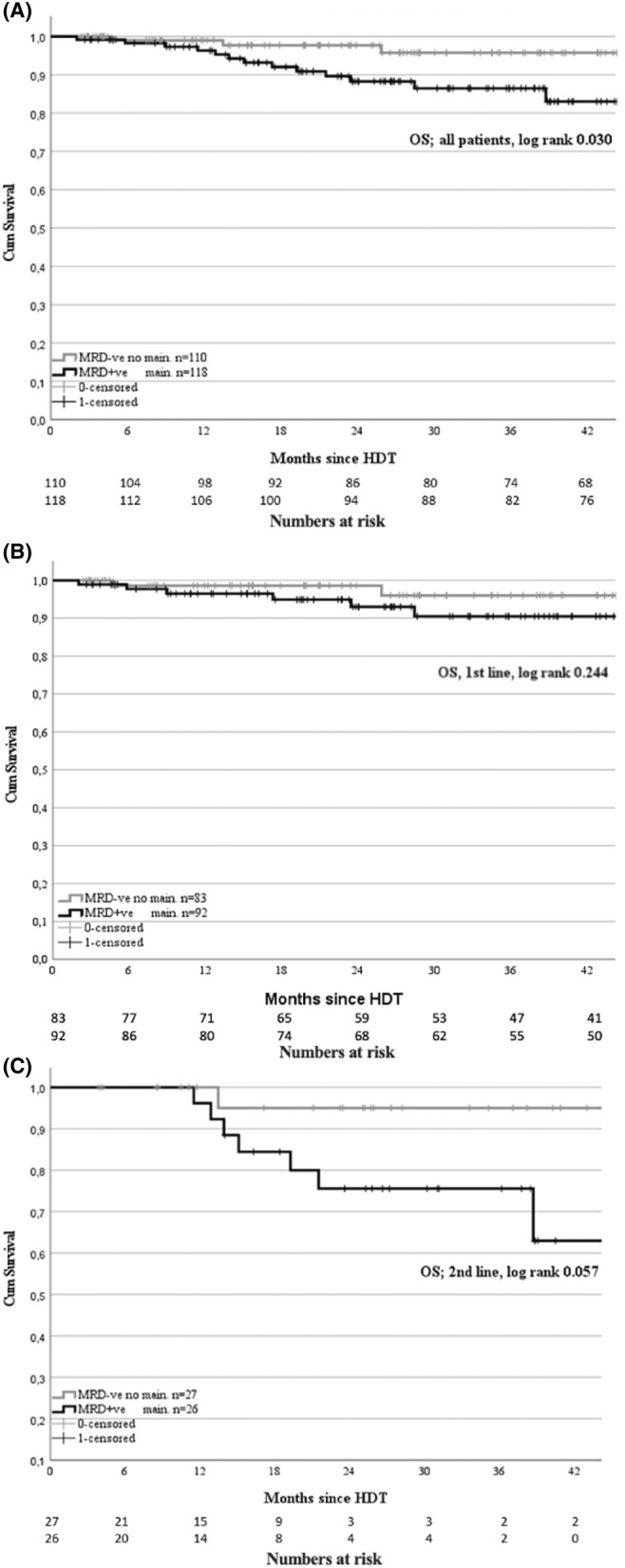
Analysis of overall survival (OS) in multiple myeloma patients grouped by minimal residual disease (MRD) status. Panel 2A; Kaplan–Meier curves of OS for all patient grouped by MRD status. Panel 2B; OS for the first‐line autologous stem cell transplantation (ASCT) patient grouped by MRD status. Panel 2C; OS for the second‐line ASCT patient grouped by MRD status.

In the examination of 3‐year OS post‐ASCT, it was observed that MRD+ patients fared worse than MRD− patients, with survival rates of 86% and 96%, respectively (*p* = 0.030, Figure 2A). Among SR patients, the 3‐year OS was 85% and 100% for MRD+ and MRD−, respectively (*p* = 0.038). However, in HR patients, no significant difference was found in the 3‐year OS between MRD+ and MRD− patients, with rates of 81% and 82%, respectively (*p* = 0.804).

When focusing on DH patients, the 3‐year OS was 80% and 100% for MRD+ and MRD−, respectively, but the difference was not statistically significant (Table 2).

For patients receiving first‐line ASCT, the 3‐year OS was 90% in MRD+ and 96% in MRD− (Figure 2B). In SR patients, the 3‐year OS was the same for MRD+ and MRD− patients (88%). For HR patients, the 3‐year OS was 78% in MRD+ and 86% in MRD− (*p* = 0.445).

Lastly, for patients treated with ASCT in the second line, the 3‐year OS was 76% in MRD+ and 95% in MRD− (*p* = 0.057, Figure 2C), and in SR patients, it was 80% in MRD+ and 100% in MRD− patients (*p* = 0.211, Table 2).

We also performed the Cox regression for the confounding factors age, gender, heavy/light chain, bone lesion, calcium, albumin, beta2microgloubilin, hemoglobin, creatinine, and high risk by FISH for OS. None of these factors affect the difference OS in any subgroup.

## 4 DISCUSSION

Post‐ASCT maintenance treatment in MM patients is a universally acknowledged standard of care (SOC) and is recommended in several international guidelines. Our study contributes to this ongoing discourse by examining maintenance treatment in a unique patient population: only minimal residual disease‐positive (MRD+) patients in first‐ and second‐line treatments following ASCT. The patients were consecutively enrolled over a 4‐year period, and while the relatively short follow‐up time, retrospective nature of the study and the smaller patient population, giving a lower statistical power compared to other published randomized trials, this study provides meaningful insights in a real‐world setting. Another limitation is that no follow‐up MRD analysis was performed neither in the MRD+ nor in the MRD− patients, which might have resulted in more insight in the effectiveness of the MRD as predictor of PFS and OS in this setting.

Our analysis corroborates the findings from the phase 3 Myeloma XI study, which demonstrated the efficacy of lenalidomide as a maintenance treatment post‐ASCT across all cytogenetic risk groups.^15^ Importantly, the MRD status did not alter the beneficial effect of lenalidomide on PFS in the Myeloma XI study, echoing the observations from our study. Furthermore, achieving MRD negativity (MRD−) after ASCT was crucial in both studies for ameliorating the poor outcomes associated with high‐risk (HR) cytogenetics.^16^

In our study, while MRD+ patients receiving maintenance treatment demonstrated no significant difference in PFS compared to MRD− patients after ASCT, the OS was still significantly poorer for MRD+ patients. These observations suggest that although maintenance therapy might improve the outcomes of MRD+ patients, it is unable to completely mitigate the negative impact of MRD+ status. A similar pattern was observed in the HR cytogenetic group, highlighting the potential value of maintenance treatment in MRD+ patients.

Response to initial therapy and achieving a prolonged initial remission duration may ultimately be the most important prognostic factor in newly diagnosed patients (NDMM) patients. There are clear data that show that achieving deep MRD− remissions can overcome high‐risk biological features and that standard‐risk patients who fail to achieve deep remissions are far worse than high‐risk ones that reach MRD−.^17^ In second‐line treated ASCT, MRD+ patients did not reach prolonged survival, the 3‐year PFS being only 27% and 28%, respectively, compared to 71% and 83% in MRD− non‐maintenance treated patients.

Minimal residual disease status continues to be investigated as a surrogate marker for OS in numerous ongoing clinical trials,^18^ and accumulating evidence underscores the pivotal role of achieving MRD− in enhancing OS and PFS.^19^ Our results reaffirm this significance, with MRD− status being the primary factor positively influencing both OS and PFS.

In conclusion, MRD status remains the most crucial predictor of outcomes in both first‐ and second‐line ASCT‐treated MM, even in the presence of maintenance treatment. MRD− status can offset the adverse effect of HR cytogenetics on PFS and OS. Our results propose that achieving MRD− status is a more potent determinant of PFS and OS than maintenance treatment and risk stratification via FISH. Given that single‐agent maintenance treatment appears insufficient, for MRD+ patients after second‐line ASCT, there is an impetus to consider more aggressive treatment strategies post‐ASCT for MRD+ patients. Future studies should aim to investigate the feasibility and effectiveness of these approaches in improving patient outcomes.

## AUTHOR CONTRIBUTIONS

**Hareth Nahi:** Conceptualization (lead); formal analysis (lead); funding acquisition (lead); investigation (lead); software (equal); writing – original draft (lead); writing – review and editing (equal). **Gabriel Afram:** Conceptualization (equal); resources (supporting); writing – original draft (supporting). **Katarina Uttervall:** Conceptualization (equal); data curation (equal); writing – original draft (supporting). **Sandra Lockmer:** Conceptualization (equal); data curation (supporting); writing – original draft (supporting). **Love Tätting:** Conceptualization (equal); writing – original draft (equal); writing – review and editing (equal). **Gösta Gahrton:** Conceptualization (equal); validation (equal); writing – original draft (equal); writing – review and editing (supporting). **Muhammad Kashif:** Conceptualization (supporting); data curation (equal); formal analysis (lead); software (lead); visualization (equal); writing – original draft (equal); writing – review and editing (equal). **Evren Alici:** Conceptualization (equal); resources (equal); writing – original draft (supporting); writing – review and editing (equal). **Olga Stromberg:** Conceptualization (equal); writing – original draft (supporting). **Monika Klimkowska:** Conceptualization (equal); writing – original draft (supporting). **Johan Lund:** Conceptualization (equal); data curation (equal); resources (equal); writing – original draft (supporting).

## FUNDING INFORMATION

This work was supported by [HN, cancerfonden, Dnr: 4‐2528/2019]. The funders had no role in the study design, data collection and analysis, decision to publish, or preparation of the manuscript.

## CONFLICT OF INTEREST STATEMENT

The authors declare no conflicts of interest.

## ETHICS STATEMENT

The study was approved by the Institutional Review Board of (EPN 2014/526‐31/3 and 2015/973‐32). All procedures performed in studies involving human participants were in accordance with the ethical standards of the institutional and/or national research committee and with the 1964 Helsinki declaration and its later amendments or comparable ethical standards.

## INFORMED CONSENT

Informed consent was obtained from all individual participants included in the study.

## Data Availability

The data that support the findings of this study are available upon reasonable request from the corresponding author. The data are not publicly available due to privacy or ethical restrictions.
